# New Appliances and Things Medical

**Published:** 1898-05-14

**Authors:** 


### NEW APPLIANCES AND THINGS MEDICAL.

[We shall be glad to receive, at our Office, 28 & 29, Southampton Street, Strand, London, W.C., from the manufacturers, specimens of all new preparations and appliances which may be brought out from time to time.]

### FERRU-COCOA.

(FERRU-COCOA MANUFACTURING COMPANY, 329, GOSWELL-ROAD, E.C.)

A sample of Ferru-Cocoa has been submitted to us by the Ferru-Cocoa Manufacturing Company. It is a preparation which is said to be made from the purest cocoa, combined with kola nut, malt, and a small quantity of the ferruginous or iron bearing elements of food. It is stated that it can be taken in any quantity, and for any length of time, and that it is not a medicine, but a true food. The results of examination of Ferru-Cocoa showed it to be a preparation resembling cocoa from which a large proportion of the natural fat of the cocoa bean has been removed, thereby rendering the preparation more digestible. It contained mineral constituents amounting to 6 per cent. The amount of iron evident in the ash was above that normally present in such preparations, and amounted in the sample examined to rather less than two-tenths of a grain of iron, expressed as Ferrous oxide, in a teaspoonful of Ferru-Cocoa. Ferru-Cocoa makes a very palatable and nutritious beverage. For all who require a larger amount of ferruginous material than is provided in ordinary articles of diet, this preparation is likely to be useful.

